# Sex-dependent relationship of polymorphisms in *CLOCK* and *REV-ERBα* genes with body mass index and lipid levels in children

**DOI:** 10.1038/s41598-023-49506-2

**Published:** 2023-12-13

**Authors:** Claudia Vales-Villamarín, Olaya de Dios, Ignacio Mahíllo-Fernández, Macarena Perales, Iris Pérez-Nadador, Teresa Gavela-Pérez, Leandro Soriano-Guillén, Carmen Garcés

**Affiliations:** 1 Lipid Research Laboratory, IIS-Fundación Jiménez Díaz, 28040 Madrid, Spain; 2 Department of Biostatistics and Epidemiology, IIS-Fundación Jiménez Díaz UAM, Madrid, Spain; 3 Department of Pediatrics, IIS-Fundación Jiménez Díaz, 28040 Madrid, Spain

**Keywords:** Genetic association study, Risk factors

## Abstract

Circadian rhythms, which are governed by a circadian clock, regulate important biological processes associated with obesity. SNPs in circadian clock genes have been linked to energy and lipid homeostasis. The aim of our study was to evaluate the associations of *CLOCK* and *REV-ERBα* SNPs with BMI and plasma lipid levels in pre-pubertal boys and girls. The study sample population comprised 1268 children aged 6–8 years. Information regarding anthropometric parameters and plasma lipid concentrations was available. Genotyping of *CLOCK* SNPs rs1801260, rs4580704, rs3749474, rs3736544 and rs4864548 and *REV-ERBα* SNPs rs2017427, rs20711570 and rs2314339 was performed by RT-PCR. The *CLOCK* SNPs rs3749474 and rs4864548 were significantly associated with BMI in girls but no in boys. Female carriers of the minor alleles for these SNPs presented lower BMI compared to non-carriers. A significant association of the *REV-ERBα* SNP rs2071570 with plasma total cholesterol, LDL-cholesterol and Apo B in males was also observed. Male AA carriers showed lower plasma levels of total cholesterol, LDL-cholesterol and Apo B levels as compared with carriers of the C allele. No significant associations between any of the studied *REV-ERBα* SNPs and plasma lipid levels were observed in females. In summary, *CLOCK* and *REV-ERBα* SNPs were associated with BMI and plasma lipid levels respectively in a sex-dependent manner. Our findings suggest that sex-related factors may interact with Clock genes SNPs conditioning the effects of these polymorphisms on circadian alterations.

## Introduction

Circadian rhythms regulate multiple aspects of physiology and metabolism^1^. These rhythms are generated by an endogenous mechanism involved in endocrine signaling^2^, which is comprised of circadian clocks^3^. This circadian clock system has been related to metabolic processes^4^ and metabolic and cardiovascular disease^5–8^. In this regard, the role of the circadian clock system regulating adipose tissue physiology^9^ and therefore energy balance and glucose and lipid metabolism has been established^10–13^.

The control of the circadian rhythms in mammals is carried out by the Clock genes that encode proteins implicated in positive and negative regulatory pathways^14^. Among them, CLOCK is a key component of the molecular circadian clock, regulating the expression of an important number of transcription factors^15^. CLOCK has been linked to metabolism homeostasis, including lipid metabolism^16^. Several studies in different racial populations have associated single nucleotide polymorphisms (SNPs) in the CLOCK gene with obesity and body mass index (BMI)^17–21^ as well as with metabolic syndrome^18,22^ and type-2 diabetes^23,24^.

Other of the components of the circadian clock that has a key role in the machinery of circadian rhythms is REV-ERBα, that regulates the circadian rhythms in a negative way. REV-ERBα suppresses the expression of the main components of this machinery ARTNL/BMAL1, CLOCK and CRY1^14^. REV-ERBα is considered to be essential regulating circadian behavior and metabolism^25,26^. Thus, SNPs in the REV-ERBα gene have been investigated in relationship with metabolic alterations and have been associated with obesity and body mass index in adults^27–29^ as well as in children population^27,30^. However, a sex-dependent association of the SNPs in the REV-ERBα gene with anthropometric variables has been described^28,30^.

The association of polymorphisms in *CLOCK* and *REV-ERBα* with blood lipid levels has been less studied. Studies analyzing *CLOCK* SNPs failed to find any association^24,31,32^. A study analyzing the implication of *CLOCK* SNPs in weight reduction reported their association with differences in plasma cholesterol in response to dietary treatment^19^. Studies analyzing the association of plasma lipid levels with *REV-ERBα* SNPs failed to find any significant association^27^. However, no differences between sexes were examined.

Our study aimed to evaluate the relationship of several SNPs in *CLOCK* (rs180126, rs4580704, rs3749474, rs3736544, rs4864548) and in *REV-ERBα* (rs2071427, rs2071570, rs2314339) with BMI and plasma lipid levels in 6–8-year-old boys and girls.

## Results

Table 1 shows characteristics of the study participants according to sex. 637 were males and 631 were females. The mean age was 7.2 ± 0.6 years, without differences between sexes. The prevalence of obesity was similar in boys and girls (8.3% and 9.8%, respectively). Compared to males, females presented significantly higher LDL-cholesterol (110.2 ± 26.9 mg/ml vs. 107.3 ± 25.4 mg/ml, *p* < 0.05) and Apo B (71.4 ± 14.9 mg/ml vs. 68.8 ± 14.1 mg/ml, *p* < 0.01) levels, and lower Apo A-I (135.6 ± 18.9 mg/ml vs. 138.3 ± 19.1 mg/ml, *p* < 0.05) levels.

**Table 1:** Characteristics (means ± SD) of the study participants by sex.

Variables	Boys (n = 637)	Girls (n = 631)	*p* value
Age (years)	7.2 ± 0.6	7.2 ± 0.7	0.733
Weight (kg)	26.8 ± 5.3	26.7 ± 5.4	0.619
BMI (kg/m^2^)	16.9 ± 2.4	17.0 ± 2.5	0.662
Total Cholesterol (mg/dl)	181.8 ± 26.2	183.6 ± 28.4	0.233
Triglycerides (mg/dl)	71.2 ± 25.4	74.2 ± 26.5	0.042
LDL-Cholesterol (mg/dl)	107.3 ± 25.4	110.2 ± 26.9	0.047
HDL-Cholesterol (mg/dl)	60.2 ± 13.1	58.8 ± 13.3	0.052
Apo B (mg/dl)	68.8 ± 14.1	71.4 ± 14.9	0.002
Apo A-I (mg/dl)	138.3 ± 19.1	135.6 ± 18.9	0.012

BMI, body mass index; LDL-Cholesterol, low-density lipoprotein cholesterol; HDL-Cholesterol, high-density lipoprotein cholesterol; Apo B, apolipoprotein B; Apo A-I, apolipoprotein A-I.

Table 2 shows the location, minor allele frequencies and HWE *p* values for the studied SNPs. The minor allele frequencies for the analyzed SNPs ranged from 27.1 to 38.5% for *CLOCK* SNPs and from 12.3 to 28.7% for *REV-ERBα* SNPS. Frequencies for the studied SNPs were consistent with Hardy–Weinberg equilibrium (*p* > 0.05).

**Table 2:** Characteristics of studied SNPs in the *CLOCK* and *REV-ERBα* genes.

Name	Location	HWE *p* value	Alleles	Minor Allele	MAF
CLOCK SNPs					
rs1801260	3′ UTR	0.3278	A/G	G	0.271
rs4580704	Intron 9	0.9748	C/G	G	0.384
rs3749474	3’ UTR	0.4638	C/T	T	0.336
rs3736544	Exon 20	0.8945	G/A	A	0.385
rs4864548	2 KB Upstream	0.4470	G/A	A	0.337
REV-ERBα SNPs					
rs2071427	Intron 1	0.4674	C/T	T	0.287
rs2071570	2 KB Upstream	0.5539	C/A	A	0.203
rs2314339	Intron 2	0.5814	C/T	T	0.123

HWE, Hardy–Weinberg Equilibrium; MAF, minor allele frequency.

### Associations of the studied SNPs with BMI

The analysis of the association between the studied CLOCK gene SNPs and BMI is shown in Table 3. The variants rs3749474 and rs4864548 were found to be significantly associated with BMI, in girls but no in boys. Carriers of the minor allele T of the SNP rs3749474 and the minor allele A of the SNP rs4864548 presented significantly lower BMI compared to non-carriers (*p* value 0.032 and 0.022, respectively). No association between BMI and the *CLOCK* SNPs rs1801260 and rs4580704 was found in any sex.

**Table 3:** BMI (kg/m^2^), expressed by means and standard deviation, according to the genotypes of the studied *CLOCK* SNPs by sex.

SNP	Genotype	N	Boys	N	Girls
*CLOCK* rs1801260	AA	322	17.0 ± 2.42	339	16.8 ± 2.45
	AG	267	16.9 ± 2.32	242	17.3 ± 2.59
	GG	44	17.4 ± 2.93	41	16.9 ± 2.55
*CLOCK* rs4580704	CC	244	17.1 ± 2.38	233	16.8 ± 2.34
	CG	291	17.0 ± 2.51	302	17.2 ± 2.66
	GG	98	16.5 ± 2.07	87	16.9 ± 2.40
*CLOCK* rs3749474	CC	286	16.9 ± 2.42	260	17.2 ± 2.58^a^
	CT	281	17.0 ± 2.38	290	**16.9 ± 2.58**
	TT	65	17.1 ± 2.51	71	**16.4 ± 1.89**
*CLOCK* rs3736544	GG	244	17.1 ± 2.39	232	16.7 ± 2.25
	GA	292	17.0 ± 2.51	300	17.2 ± 2.72
	AA	97	16.5 ± 2.06	90	16.9 ± 2.39
*CLOCK* rs4864548	GG	285	16.8 ± 2.40	261	17.3 ± 2.62^b^
	GA	283	17.0 ± 2.39	289	**16.9 ± 2.53**
	AA	65	17.1 ± 2.51	71	**16.4 ± 1.89**

^a^*p* value < 0.05, comparing BMI in carriers of the minor allele T of the SNP rs3749474 versus BMI in non-carriers.

^b^*p* value < 0.05, comparing BMI in carriers of the minor allele A of the SNP rs4864548 versus BMI in non-carriers.

The analysis of the association between the analyzed *REV-ERBα* SNPs and BMI revealed no significant associations.

### Associations of the studied SNPs with plasma lipid levels

**Figure 1:**
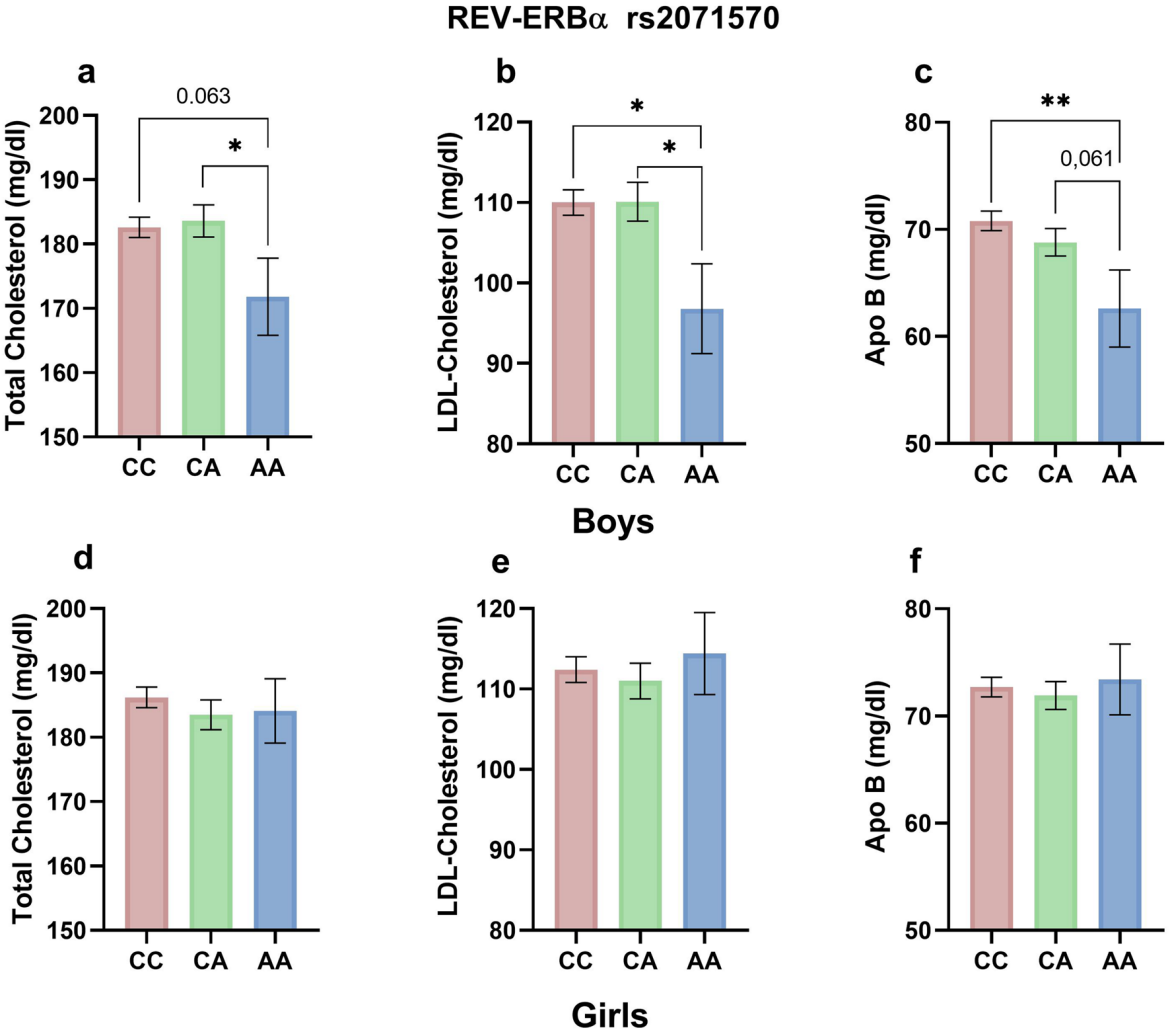
Plasma lipid levels according to genotypes of the *REV-ERBα* rs2071570 SNP in boys (**a**–**c**) and girls (**d**–**f**). *p value < 0.05; **p value < 0.01.

When analyzing the association of these SNPs in *CLOCK* and in *REV-ERBα* with plasma lipid levels, we observed, in males, a significant association of the SNP in *REV-ERBα* rs2071570 with total cholesterol (Fig. 1a), LDL-cholesterol (Fig. 1b) and Apo B (Fig. 1c) that is not observed in females (Fig. 1d,e,f). Boys homozygous for the minor allele A showed lower total cholesterol levels compared to carriers of the major allele C (AA vs. CA *p* = 0.053) (Fig. 1a). Significantly lower plasma levels of LDL-cholesterol were observed in male carriers of the AA allele when comparing with carriers of the C allele (AA vs. CC *p* = 0.021; AA vs. CA *p* = 0.027) (Fig. 1b). Similar results were found for Apo B levels (Fig. 1c). AA males showed lower plasma levels of Apo B as compared with homozygous for the C allele (*p* = 0.009) and as compared with CA carriers (*p* = 0.061). No associations with plasma lipid levels were found for the rest of SNPs analyzed in CLOCK and REV-ERBα genes in any sex.

## Discussion

In this study we have analyzed the potential associations of SNPs of the CLOCK and REV-ERBα genes with body mass index (BMI) and lipid levels in a cohort of boys and girls aged 6–8 years. We found sex-dependent associations of the SNPs rs3749474 and rs4864548 of *CLOCK* with BMI and of the SNP rs20711570 of *REV-ERBα* with plasma total cholesterol, LDL-cholesterol and Apo-B.

The association between SNPs of the CLOCK gene and anthropometric variables has been widely investigated. In our cohort, we found that carriers of the less common alleles for both, the rs3749474 and rs4864548 *CLOCK* SNPs, presented lower BMI compared to non-carriers. These SNPs, alone or combined in haplotypes, have been linked to the individual susceptibility to obesity in adults^17–21^. However, we found no association with rs1801260, one of the *CLOCK* SNPs most frequently studied in relationship with anthropometric variables in adult populations. Studies conducted in Caucasian populations have been able to find consistent associations of the SNP rs1801260 with obesity and BMI^19,20,22,24,33,34^ that are not evident in our population. Furthermore, the associations between the *CLOCK* SNPs and BMI found in our study are presented in girls but no in boys. A sex-dependent association between *CLOCK* SNPs and overweight and obesity has been previously described in adults^35^. We hypothesized that age-related factors may be affecting the relationship of these SNPs with anthropometric variables. It has been described that aging may result in gain or loss of rhythmic circadian expression^36^. In this sense, a different regulation of circadian gene expression between middle/older aged individuals and younger adults has been reported^37^ which may contribute to explain the described discrepancies in findings depending on age.

Regarding the relationship of *CLOCK* SNPs with plasma lipid levels, no consistent associations have been found in our cohort. The relationship of *CLOCK* SNPs with plasma lipid levels remains imprecise. No association was found in the PREDIMED trial analyzing the association of the SNP of *CLOCK* rs4580704 with total cholesterol, LDL-cholesterol or HDL-cholesterol^24^. However, a study analyzing the implication of Clock genes in weight reduction in obese patients found that *CLOCK* SNPs were associated with serum cholesterol changes in response to a dietary intervention^19^. Unlike this lack of association with lipid levels observed for *CLOCK* SNPs, we found an association of plasma total cholesterol, LDL-cholesterol and Apo B levels with the SNP rs20711570 in the promoter region of the *REV-ERBα*, which is another important regulator of metabolism. Again, we described a sex-dependent association of this SNP with lipid parameters, as we observed that male carriers of the AA genotype showed significantly lower plasma levels of total cholesterol, LDL-cholesterol and Apo B concentrations as compared with C allele carriers. However no significant differences in lipid levels among genotypes were observed in females. *REV-ERBα* polymorphisms have been studied in relationship with body weight regulation, but its association with lipid levels has been less explored. An association of genetic variants in the REV-ERBα gene, including the *REV-ERBα* SNP rs2071570, with obesity has been reported^27,28,30^. However, as happened with *CLOCK* SNPs, this association appears to be sex-dependent^28^. In a study including adolescents, Nascimento Ferreira et al. described a significant association between *REV-ERBα* SNPs and BMI only in boys^30^. In our study, although we failed to find any association of the *REV-ERBα* SNPs with BMI, we observed a sex-dependent association of the SNP rs20711570 of *REV-ERBα* with lipid levels, which reinforces the hypothesis of the existence of sex-related factor interacting with these Clock genes polymorphisms affecting metabolic alterations. In this sense, a sexual dimorphism in Clock genes expression in human adipose tissue has been described^38^. The role of sex in the different influence of circadian rhythms on cardiovascular disease in males and females has been extensively discussed^39^. In this regard, the sex-related differences in Clock controlled processes have been associated with suprachiasmatic nucleus (SCN) morphology and signaling^40^, as sex differences in SCN morphology and neuropeptide expression are known^41^. SCN receives both estrogenic and androgenic inputs^42^. These aspects may be related to the different association of the studied SNPs with anthropometric and lipid parameters observed in males and females.

More studies are needed taking into account how the possible interactions between the Clock genes polymorphisms and sex, as a biological variable, influence the development of obesity and metabolic alterations, including variations in lipid levels^43^.

In conclusion, in our study we report sex-dependent associations between the *CLOCK* SNPs rs3749474 and rs4864548 and BMI as well as between the *REV-ERBα* SNP rs20711570 and plasma LDL-cholesterol and Apo-B concentrations. Our findings suggest the role of the interaction between sex-related factors and Clock genes polymorphisms in obesity and plasma lipid levels.

## Materials and methods

### Study participants

Our population-based sample comprises a total of 1268 prepubertal children (6–8-year-old), participants in a cross-sectional study examining cardiovascular risk factors in Spanish schoolchildren^44^ in whom information on anthropometric variables and lipid levels was available.

The study complies with Helsinki Declaration guidelines and was approved by the Clinical Research Ethics Committee of the IIS-Fundación Jiménez Díaz (PIC016-2019 FJD). All parents provided written informed consent for their children to participate in the study. Children reported by their parents to be suffering from chronic diseases were excluded of the study.

### Anthropometric measurements

Weight and height were measured with children wearing light clothing and barefoot. Height was measured to the millimeter using a portable stadiometer. Weight was recorded to the nearest 0.1 kg using a standard electronic digital scale. The classification by obesity category was carried out according to the cut-off points established for children by Cole et al.^45^.

### Biochemical determinations

Fasting (12 h) blood samples were obtained by venipuncture. Serum and plasma samples were separated by centrifugation and stored at − 70 °C. Biochemical determinations were performed as previously described^44^. Cholesterol and triglyceride (TG) concentrations were measured enzymatically (Menarini Diagnostics, Florence, Italy) in a RA-1000 Autoanalyzer (Technicon Ltd., Dublin, Ireland). High-density lipoprotein cholesterol (HDL-cholesterol) was measured after precipitation of apolipoprotein B-containing lipoproteins with phosphotungstic acid and Mg (Roche Diagnostics, Madrid, Spain). Plasma apolipoprotein A-I (Apo A-I) and apolipoprotein B (Apo B) concentrations were measured by immunonephelometry (Dade Berhing, Frankfurt, Germany). Low-density lipoprotein cholesterol (LDL-cholesterol) was calculated according to the Friedewald formula.

### SNP genotyping

The SNPs of *CLOCK* rs1801260, rs4580704, rs3749474, rs3736544 andrs4864548 and the SNPs of *REV-ERBα* rs2017427, rs20711570 and rs2314339 SNPs were selected to be studied in our population based on the results of previous studies reporting association of these SNPs with anthropometric variables. Genotyping was carried by RT-PCR using a 7500 Fast Real-Time System (Applied Biosystems) and performed with 10 ng of genomic DNA. Samples were cycled under the following recommended conditions: 95 °C for 10 min, 95 °C for 15 s, and 60 °C for 1 min, repeated over 40 cycles.

The SNPs of *CLOCK* rs1801260, rs4580704, rs3749474, rs3736544 and rs4864548 were genotyped using the C_8746719_20, C_28028791_10, C_26405955_10, C_22273263_10 and C_11821276_10 Applied Biosystems predesigned allelic discrimination TaqMan® assays. The SNPs of *REV-ERBα* rs2017427, rs20711570 and rs2314339 using the TaqMan® assays C_7479334_10, C_7479332_10, and C_177490_10 respectively (Applied Biosystems).

### Statistical analyses

Participant characteristics were described by means and standard deviations for continuous variables and counts and percentages for categorical variables. Comparisons between boys and girls were performed using the Student’s t test. Hardy–Weinberg deviations for each of the studied SNPs were tested by a chi-squared goodness-of-fit test. The normality of the distribution of the variables under study was examined using the Kolmogorov–Smirnov test. Differences in mean values for the variables under study according to the different genotypes of each of the SNPs studied were tested individually using the linear regression models taking the more common genotype for each polymorphism as reference. Means and standard deviations of the analyzed variables corresponding to each genotype are shown in Table 3 and means and standard error are shown in Fig. 1. Analyses were performed separately in males and females. Statistical analysis was performed using the R software, 4.1.2 (R: A language and environment for statistical computing. R Foundation for Statistical Computing, Vienna, Austria) and GraphPad Prism statistical software (San Diego, CA, USA, Version 8).

## Data Availability

All data analyzed during this study are included in this published article.
